# Risk prediction models for diabetic nephropathy among type 2 diabetes patients in China: a systematic review and meta-analysis

**DOI:** 10.3389/fendo.2024.1407348

**Published:** 2024-07-03

**Authors:** Wenbin Xu, Yanfei Zhou, Qian Jiang, Yiqian Fang, Qian Yang

**Affiliations:** School of Nursing, Chengdu Medical College, Chengdu, Sichuan, China

**Keywords:** type 2 diabetes, diabetic kidney disease, risk prediction model, systematic review, meta-analysis

## Abstract

**Objective:**

This study systematically reviews and meta-analyzes existing risk prediction models for diabetic kidney disease (DKD) among patients with type 2 diabetes, aiming to provide references for scholars in China to develop higher-quality risk prediction models.

**Methods:**

We searched databases including China National Knowledge Infrastructure (CNKI), Wanfang Data, VIP Chinese Science and Technology Journal Database, Chinese Biomedical Literature Database (CBM), PubMed, Web of Science, Embase, and the Cochrane Library for studies on the construction of DKD risk prediction models among type 2 diabetes patients, up until 28 December 2023. Two researchers independently screened the literature and extracted and evaluated information according to a data extraction form and bias risk assessment tool for prediction model studies. The area under the curve (AUC) values of the models were meta-analyzed using STATA 14.0 software.

**Results:**

A total of 32 studies were included, with 31 performing internal validation and 22 reporting calibration. The incidence rate of DKD among patients with type 2 diabetes ranged from 6.0% to 62.3%. The AUC ranged from 0.713 to 0.949, indicating the prediction models have fair to excellent prediction accuracy. The overall applicability of the included studies was good; however, there was a high overall risk of bias, mainly due to the retrospective nature of most studies, unreasonable sample sizes, and studies conducted in a single center. Meta-analysis of the models yielded a combined AUC of 0.810 (95% CI: 0.780–0.840), indicating good predictive performance.

**Conclusion:**

Research on DKD risk prediction models for patients with type 2 diabetes in China is still in its initial stages, with a high overall risk of bias and a lack of clinical application. Future efforts could focus on constructing high-performance, easy-to-use prediction models based on interpretable machine learning methods and applying them in clinical settings.

**Registration:**

This systematic review and meta-analysis was conducted following the Preferred Reporting Items for Systematic Reviews and Meta-Analyses (PRISMA) statement, a recognized guideline for such research.

**Systematic review registration:**

https://www.crd.york.ac.uk/prospero/, identifier CRD42024498015.

## 1 Introduction

The “Global Diabetes Map (10th Edition)” released by the International Diabetes Federation in 2021 indicates that the global number of diabetes cases has climbed to 537 million, a more than 16% increase compared to 2019 (1). By 2045, it is projected that the global diabetes patient population will reach 783 million, with China’s number expected to exceed 174 million (2). The situation regarding diabetes incidence is not optimistic. Particularly, type 2 diabetes mellitus (T2DM) accounts for 90% to 95% of all diabetes cases (3). With the increasing prevalence of T2DM, the incidence of diabetic kidney disease (DKD) has significantly risen and has become a leading cause of end-stage renal disease (4). Despite this, the awareness of DKD among diabetic patients in China remains below 20%, and the early treatment rate is less than 50% (5), indicating an urgent need to enhance the dissemination of related healthcare knowledge among diabetic patients. Studies have shown that the onset of DKD is influenced by multiple risk factors, making the early detection and screening of high-risk patients crucial (6).

Risk prediction models can analyze various risk factors of a disease and assign corresponding weights to each factor to calculate the probability or risk of future disease onset, providing a reference for healthcare professionals (7, 8). By constructing DKD risk prediction models and conducting risk assessments, the onset of kidney disease can be effectively prevented (9). In recent years, many scholars have dedicated efforts to developing risk prediction models for kidney disease in patients with T2DM. This paper aims to systematically evaluate the existing DKD risk prediction models for patients with T2DM in China, hoping to provide useful references for future scholars in model development and application.

## 2 Article type

This study is a systematic review and meta-analysis of predictive models for the risk of diabetic nephropathy in Chinese patients with type 2 diabetes.

## 3 Materials and methods

### 3.1 Inclusion and exclusion criteria for literature

The inclusion criteria were as follows: 1) study subjects aged ≥18 years; 2) participants confirmed as Chinese patients with type 2 diabetes mellitus according to diabetes diagnostic guidelines or expert consensus; 3) participants diagnosed with diabetic kidney disease according to the diagnostic guidelines for diabetic kidney disease or expert consensus; 4) studies focused on the construction and validation of risk prediction models for diabetic kidney disease in patients with type 2 diabetes mellitus; 5) study types include cohort studies, cross-sectional studies, and case–control studies; and 6) articles written in Chinese or English.

The exclusion criteria were as follows: 1) studies that only analyzed risk factors without establishing a risk prediction model; 2) studies with incomplete data or where the original texts are inaccessible; 3) reviews, systematic reviews, and meta-analyses; and 4) conference papers.

### 3.2 Literature search strategy

**Figure 1:**
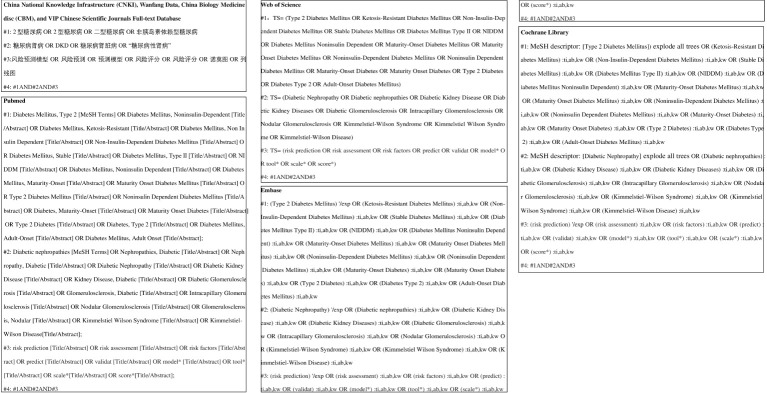
Literature search strategy.

A systematic search was conducted in PubMed, Web of Science, the Cochrane Library, Embase, China National Knowledge Infrastructure (CNKI), Wanfang Data, Chinese Biomedical Literature Database (CBM), and VIP Chinese Scientific Journals Full-text Database for studies on the construction and validation of risk prediction models for diabetic kidney disease among Chinese patients with type 2 diabetes mellitus. The search was limited to articles published up to 28 December 2023 and included both Chinese and English languages. The search strategy combined the use of MeSH terms and free words, supplemented by manual searches to include references traced back. The search terms included diabetes mellitus, type 2, type 2 diabetes, diabetic nephropathy, diabetic nephropathies, diabetic kidney disease, risk prediction, risk assessment, risk factors, predict, validate, model*, tool*, scale*, and score*. The specific search strategy is shown in **Figure 1**.

In our systematic review, we employed the PICOTS framework as recommended by the Checklist for Critical Appraisal and Data Extraction for Systematic Reviews of Prediction Modelling Studies (CHARMS) (10). This framework assists in defining the review’s objectives, search strategy, and criteria for including and excluding studies:

P (Population): patients with type 2 diabetes.
I (Index prognostic model): Risk prediction models for diabetic nephropathy among type 2 diabetes patients in China.
C (Comparative model): No competing model.
O (Outcome): The outcome focused on the occurrence of diabetic nephropathy.
T (Timing): The outcome was predicted after evaluating the personal basic information and laboratory indicators of patients with type 2 diabetes.
S (Setting): The role of the risk prediction model is to predict the probability of developing diabetic nephropathy based on the individual circumstances of patients with type 2 diabetes, thereby facilitating the implementation of preventive measures to prevent adverse events.

### 3.3 Literature selection and data extraction methods

Two researchers, trained in evidence-based courses, independently screened the literature, eliminating duplicate publications of titles and abstracts. Selection was based on the inclusion and exclusion criteria, followed by cross-checking. In case of disagreement, a third researcher was consulted to make a decision, ultimately determining the included studies.

The CHARMS checklist was first proposed in 2014 by Douglas G. Altman of the University of Oxford and Karel G. M. Moons of the University of Utrecht, among others. It is designed to clarify the issues of systematic reviews and analyze the methodology and quality of original studies (10). Researchers used the CHARMS checklist to formulate a data extraction form for extracting characteristics of the literature. Specific items extracted included the first author, year of publication, type of study, modeling methods, validation methods, calibration methods, predictive performance, predictive factors, etc. (11). Two researchers independently extracted the information from the literature, and in case of disagreement, a third researcher was consulted for resolution.

### 3.4 Risk of bias and applicability Evaluation

The Prediction Model Risk of Bias Assessment Tool (PROBAST) was developed in 2019 by Wolff and colleagues as a specialized tool for assessing studies that develop or validate diagnostic or prognostic multifactorial prediction models (12). The risk of bias assessment covers four domains: participants, predictors, outcome, and analysis. Each domain includes two to nine key questions, answered with “yes/probably yes,” “no/probably no,” or “no information,” corresponding to the judgment of “low risk of bias,” “high risk of bias,” or “unclear.” If all questions in a domain are judged as “low risk of bias,” that domain is considered to have a “low risk of bias.” If one or more questions are judged as “high risk of bias” or “unclear,” the domain is considered to have a “high risk of bias” or to be “unclear.” The applicability assessment covers three domains: participants, predictors, and outcomes, excluding the key questions, with evaluation outcomes similar to those of the risk of bias (13, 14). Two researchers used the PROBAST tool to assess the risk of bias and applicability of the included prediction model studies. In cases of disagreement, a third researcher was consulted for a decision.

### 3.5 Statistical analysis

In this study, STATA 14.0 software was used to perform meta-analysis. Heterogeneity among studies was evaluated using the Higgins *I*² statistic. An *I*² value of ≤25% indicates low heterogeneity, 25% < *I*² ≤ 50% indicates moderate heterogeneity, and *I*² > 50% indicates high heterogeneity. If *P* > 0.05 and *I*² ≤ 50%, the heterogeneity is considered acceptable, and a fixed-effect model is used; otherwise, a random-effects model is employed to combine the effect sizes. Furthermore, the area under the curve (AUC) of the receiver operating characteristic (ROC) curve is used as the measure of effect size, with specific values and 95% confidence intervals (CI) provided. An AUC range of 0.7 to 0.9 indicates moderate predictive accuracy, while an AUC > 0.9 suggests high diagnostic accuracy. A *P* < 0.05 is considered statistically significant. The Egger’s test was used to identify publication bias. If the *P*-value is greater than 0.05, it indicates a low likelihood of publication bias. Finally, a funnel plot was created (15). Funnel plot inspection is a commonly used statistical tool in meta-analysis to assess the reliability and consistency of the study results. It visually represents the relationship between effect size and study size, helping researchers identify potential publication bias and other forms of heterogeneity. In a funnel plot, each small dot represents a study or a set of data. The horizontal axis measures the study results (such as effect size or sample size), while the vertical axis represents each study’s weight or confidence (such as standard error or sample variance). Ideally, if all studies are accurate and unbiased, these dots should form a symmetrical funnel shape, with small sample studies scattering around larger sample studies, which typically show smaller effect sizes and tighter clustering. An asymmetrical or skewed funnel plot may indicate the presence of publication bias, where small sample studies (possibly because they are more likely to report extreme effect sizes) are missing on one side of the plot. Alternatively, this asymmetry might be due to other factors like selective reporting or methodological quality differences, which distort the relationship between effect size and study size. When a funnel plot shows asymmetry, researchers need to investigate the possible causes further and consider the impact of these factors on the study conclusions.

## 4 Results

### 4.1 Literature search process and results

**Figure 2:**
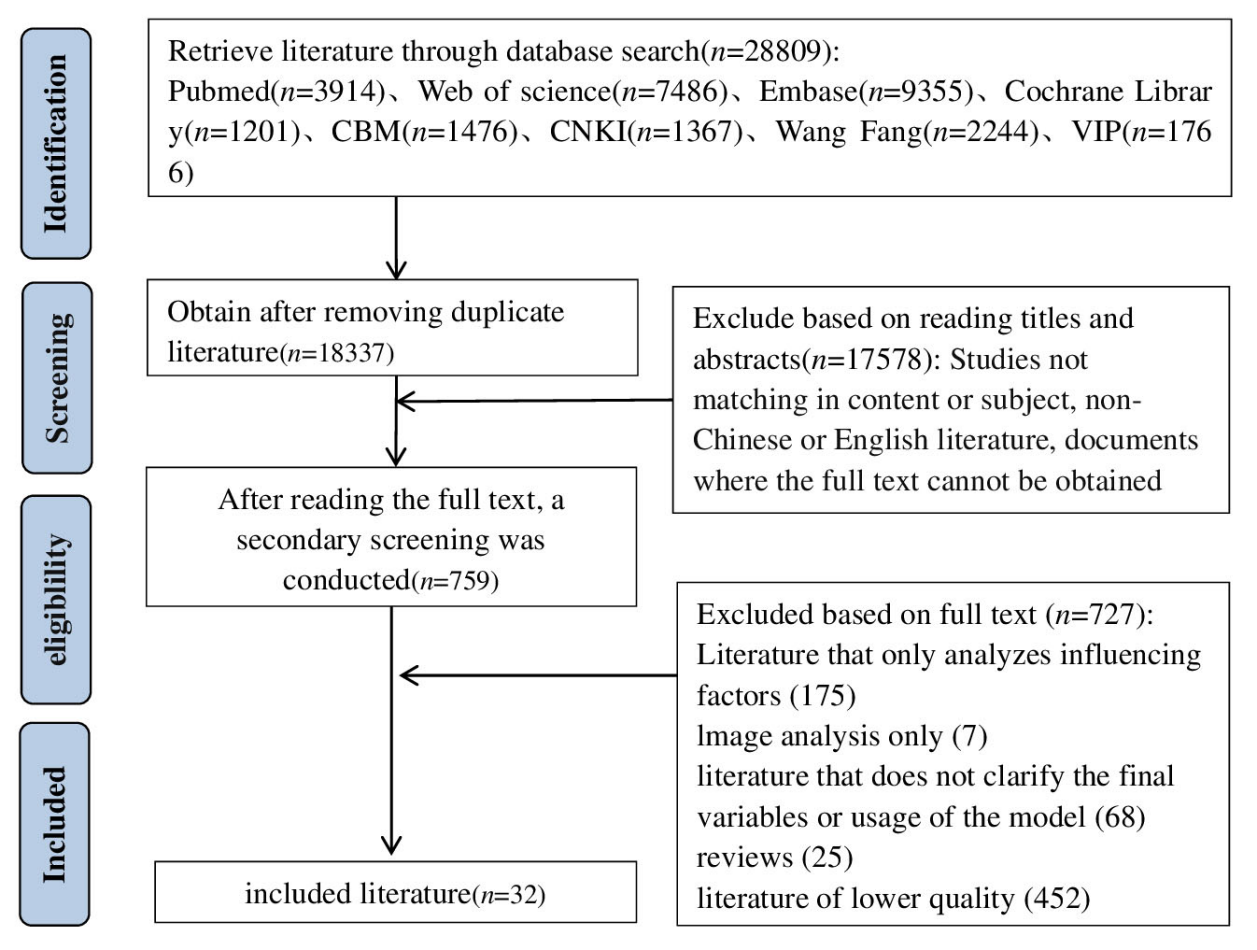
Literature screening process.

The database search yielded a total of 28,809 articles. After removing duplicates using the Note Express software, 18,337 articles remained. Subsequent screening of titles and abstracts reduced the pool to 759 articles. After a full-text review, 32 articles were ultimately included in the study (16–47). **Figure 2** shows the Preferred Reporting Items for Systematic Reviews and Meta-Analyses (PRISMA) flow diagram, illustrating the comprehensive literature screening process and results.

### 4.2 Characteristics of the included studies

Among the 32 studies included, 12 were published domestically (16–25), and 20 were published internationally (26–47). The most contributions came from scholars in Jiangsu Province, with a total of seven studies (18, 20, 28, 30, 38, 40, 41). The earliest studies were published in 2017 (35, 40), and there were 23 studies published in the last three years (18, 20, 21, 23–32, 35, 37–39, 41, 43, 45, 46). In terms of study types, there were 10 case–control studies (16, 17, 21, 24, 25, 30, 33, 36, 45, 47), 5 cross-sectional studies (19, 22, 34, 35, 39), and 16 cohort studies (18, 20, 23, 27–29, 31, 32, 37, 38, 40, 41, 43, 44, 46, 47). The specific characteristics of the included studies are detailed in **Table 1**.

**Table 1:** Basic characteristics of the included literature.

Author	Year of publication	Country of the author	Location of research	Study type
Nan (22)	2019	Beijing	Dongzhimen Hospital affiliated with Beijing University of Chinese Medicine, Dongfang Hospital affiliated with Beijing University of Chinese Medicine and 15 other hospitals	Cross-sectional study
Wang et al. (19)	2019	Zhengzhou City, Henan Province	Six community health service centers	Cross-sectional study
Yang et al. (16)	2020	Tangshan City, Hebei Province	Affiliated Hospital of North China University of Science And Technology	Retrospective case–control study
Hou et al. (17)	2020	Qingdao City, Shandong Province	Affiliated Hospital of Qingdao University	Retrospective case–control study
Lu et al. (23)	2021	Xi’an City, Shanxi Province	The First Affiliated Hospital of Air Force Military Medical University	Retrospective cohort study
Zhang et al. (18)	2021	Suzhou City, Jiangsu Province	Nanjing Medical University Affiliated Suzhou Hospital	Retrospective cohort study
Gao (20)	2022	Nanjing City, Jiangsu Province	Li’s United Clinic	Retrospective cohort study
Zhang et al. (25)	2023	Beijing	Capital Medical University affiliated Beijing Friendship Hospital	Retrospective case–control study
Cao et al. (21)	2023	Shijiazhuang City, Hebei Province	Shijiazhuang Second Hospital	Retrospective case–control study
Han et al. (24)	2023	Urumqi City, Xinjiang Uygur Autonomous Region	Xinjiang Medical University First Affiliated Hospital	Retrospective case–control study
Wu et al. (44)	2017	Shanghai	Shanghai Sixth People’s Hospital	Retrospective cohort study
Miao et al. (40)	2017	Huai’an City, Jiangsu Province	Jiangsu provincial center for disease control and Prevention	Retrospective cohort study
Liao et al. (36)	2019	Taizhong City, Taiwan Province	China University of Medicine, Taiwan Province	Retrospective case–control study
Hu et al. (34)	2020	Shanghai	Six community health service centers	Cross-sectional study
Jiang et al. (33)	2020	Beijing	China-Japan Friendship Hospital	Retrospective case–control study
Wang et al. (27)	2021	Zhengzhou City, Henan Province	The First Affiliated Hospital of Zhengzhou University	Retrospective cohort study
Dong et al. (31)	2021	Hong Kong Special Administrative Region	Hong Kong Pharmaceutical Administration	Retrospective cohort study
Xi et al. (45)	2021	Guilin City, Guangxi Province	The Affiliated Hospital of Guilin Medical College	Retrospective case–control study
Xu et al. (46)	2021	Jinhua City, Zhejiang Province	Zhejiang University Medical College Affiliated Jinhua Hospital	Retrospective cohort study
Yang et al. (39)	2022	Urumqi City, Xinjiang Uygur Autonomous Region	Xinjiang Medical University First Affiliated Hospital	Cross-sectional study
Dong et al. (29)	2022	Beijing	The General Hospital of the People’s Liberation Army	Retrospective cohort study
Sun et al. (30)	2022	Xuzhou City, Jiangsu Province	Xuzhou Central Hospital	Retrospective case–control study
Wu et al. (35)	2022	Changsha City, Hunan Province	The Third Xiangya Hospital of Central South University	Cross-sectional study
Sun et al. (37)	2022	Haikou City, Hainan Province	Li’s United Clinic	Retrospective cohort study
Zhou et al. (38)	2022	Xuzhou City, Jiangsu Province	Affiliated Hospital of Xuzhou Medical University, Xuzhou Central Hospital and other four hospitals	Retrospective case–control study
Gao et al. (41)	2022	Nanjing City, Jiangsu Province	Zhongda Hospital affiliated to Southeast University, First Affiliated Hospital of Zhengzhou University	Retrospective cohort study
Zhang et al. (42)	2022	Beijing	China-Japan Friendship Hospital	Retrospective cohort study
Hui et al. (43)	2022	Taiyuan City, Shanxi Province	Shanxi Provincial People’s Hospital	Retrospective cohort study
Gao et al. (26)	2023	Wuhan City, Hubei Province	Wuhan University Zhongnan Hospital	Retrospective cohort study
Mu et al. (28)	2023	Changzhou City, Jiangsu Province	The Third Affiliated Hospital of Soochow University	Retrospective cohort study
Liu et al. (32)	2023	Chongqing	The Affiliated Hospital of Chongqing Medical University	Retrospective cohort study
Hui et al. (47)	2023	Taiyuan City, Shanxi Province	Shanxi Provincial People’s Hospital	Retrospective case–control study

### 4.3 Construction methods and predictive efficacy of the included models

The sample sizes of the studies included ranged from 102 to 141,516, with the incidence of diabetic nephropathy in patients with type 2 diabetes ranging from 6.0% to 62.3%. Modeling methods included logistic regression analysis, LASSO regression, Cox regression, machine learning, and R language. The range of the area under the curve (AUC) for the models was from 0.713 to 0.949. A total of 31 studies (16–24, 26–47) conducted internal validation. Specific details on the model construction methods and predictive efficacy of the included studies are provided in **Table 2**.

**Table 2:** Model construction method and predictive performance.

Included literature	Modeling dataset	Validation dataset	IDH incidence rate	Validation method	Modeling methods	Internal validation methods	Model presentation format	Model performance	
								AUC	Calibration
Nan	508	55	42.7%	A	C	S	Model equation	0.747 (0.687∼0.808)	–
Wang et al.	461	197	20.9%	A	C	S	Risk assessment form	0.673 (0.592∼0.753)	–
Yang et al.	101	101	31.1%	A	R	T	Nomograms	0.943	X
Hou et al.	559	559	49.9%	A	C	T	Nomograms	0.852 (0.822∼0.882)	W, X
Lu et al.	933	378	15.3%	A	C, D	S	Nomograms	0.762 (0.734∼0.789)	W, X
Zhang et al.	873	873	55.7%	A	C	T	Nomograms	0.904 (0.880∼0.928)	X
Gao	4,228	1,812	30.0%	A	I	S	Neural network	0.803	–
Zhang et al.	200	200	38.0%	–	C	–	Nomograms	–	–
Cao et al.	200	200a/200b	–	A, B	C	S	–	0.949 (0.922∼0.968)	W, X
Han et al.	641	321	8.3%	A	C, D	S	Nomograms	0.866 (0.839∼0.894)	W, X
Wu et al.	4,795	4,795a/3,515b	12.3%	A, B	C	T	Risk assessment form	0.713 (0.692∼0.734)	W
Miao et al.	5,705	6,066	–	A	E	S	–	–	X
Liao et al.	246	246a/179b	28.1%	A, B	C	T	Risk assessment form	0.78 (0.75∼0.81)	W
Hu et al.	3,489	3,489	20.1%	A	C, D	T	Nomograms	0.744 (0.724∼0.764)	W, X
Jiang et al.	214	214a/88b	47.4%	A, B	C, D	T	Nomograms	–	W
Wang et al.	2,163	2,163	9.0%	A	C	V	Nomograms	0.808 (0.782∼0.834)	W, X
Dong et al.	94,250	47,266	6.0%	A	E	S	Nomograms	–	W, X
Xi et al.	1,095	1,095	18.5%	A	C, D	T	Nomograms	0.813 (0.778∼0.848)	X
Xu et al.	213	213	–	A	C	U	Nomograms	0.783 (0.709∼0.856)	X
Yang et al.	521	521a/185b	27.6%	A, B	C	T	Nomograms	0.773 (0.726∼0.821)	X
Dong et al.	652	164	–	A	C, F*, H, K, L, M, N	S	Machine learning model	0.815 (0.747∼0.882)	–
Sun et al.	14,628	4,876	14.3%	A	C	S	Nomograms	0.895	–
Wu et al.	437	291	39.3%	A	C, H, J*	S	Machine learning model	0.884	–
Sun et al.	4,455	4,455a/2,504b	34.2%	A, B	C	S	Risk assessment form	0.659 (0.636∼0.681)	W
Zhou et al.	102	102	–	A	C	V	Nomograms	0.941 (0.898∼0.984)	W, X
Gao et al.	307	307a/206b	–	A, B	E	S	Nomograms	0.793 (0.746∼0.840)	X
Zhang et al.	629	314	25.7%	A	C, D	S	Nomograms	0.871 (0.83∼70.902)	W, X
Hui et al.	241	241	20.7%	A	C, D, G*, H, J	U	Machine learning model	0.825	–
Gao et al.	328	100	62.3%	A	J	U	Machine learning model	0.874 (0.870∼0.890)	–
Mu et al.	365	156	24.7%	A	D	T	Nomograms	0.826 (0.775∼0.876)	X
Liu et al.	2,899	725	51.2%	A	C, D, H, J, N, O*, P, Q	V	Machine learning model	0.861	–
Hui et al.	780	178	34.0%	A	C	S	Risk assessment form	0.876 (0.825∼0.928)	W, X

A = internal validation Set; B = external validation set; C = logistic regression; D = LASSO regression; E = Cox regression; F = decision tree; G = support vector machine; H = XGboost; I = LSTM neural network; J = random forest; K = gradient boosting machine (GBM); L = AdaBoost; M = artificial neural network; N = light GBM; O = CatBoost; P = extra tree classifier; Q = gradient boosting; R = R language; S = K-fold cross-validation; T = bootstrap; U = five-fold cross-validation; V = 10-fold cross-validation; W = Hosmer–Lemeshow test; X = calibration curve; *= optimal model.

### 4.4 Predictive factors of the included models

**Figure 3:**
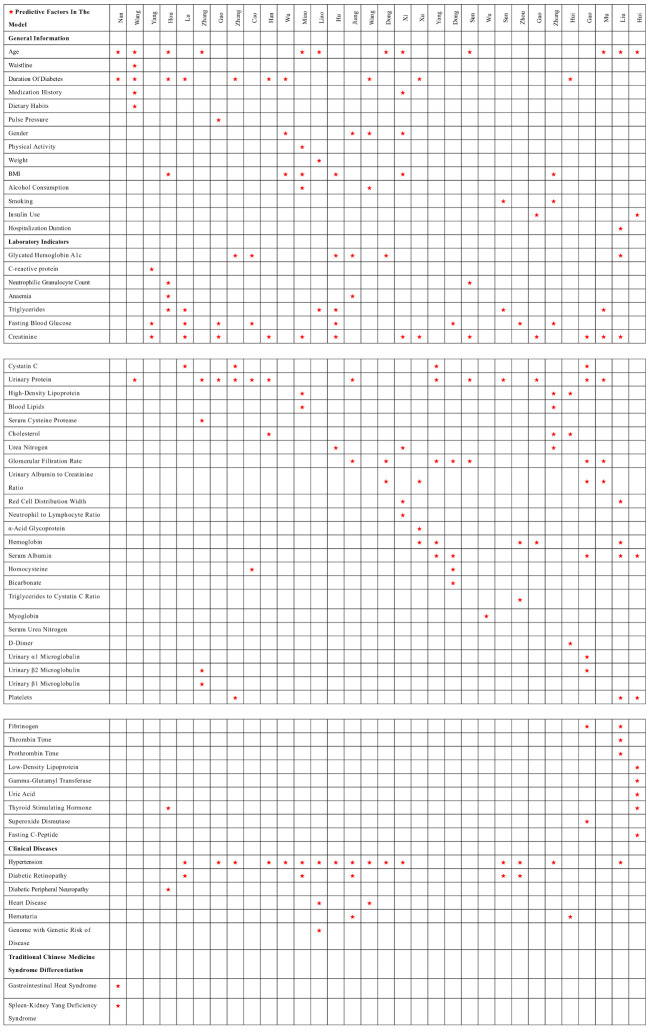
Distribution of predictor variables in the model. ☆: Predictive factors In the model.

Among the 32 studies, 30 reported the presentation form of the prediction models, mainly divided into nomograms, model equations, machine learning models, risk score charts, and neural networks, among others. The most frequently occurring predictive factors were hypertension, urinary protein, creatinine, duration of diabetes, age, and fasting blood glucose. The distribution of predictive factors for each model can be seen in **Figure 3**.

### 4.5 Methodological quality assessment results of the included studies

#### 4.5.1 Domain of the study participants

The bias related to study participants primarily stems from the type of study conducted. Twenty-seven studies (16–18, 21, 23–33, 35–47), which were based on retrospective case–control or cohort studies, were judged to be at high risk of bias. Other studies related to the study participants were considered to have a lower bias.

#### 4.5.2 Domain of the predictive factors

Regarding predictive factors, the 32 studies maintained consistent definitions and assessment methods for the predictive factors used across different study populations. When evaluating predictive factors, the researchers were blinded to outcome information, ensuring that all included predictive factors were statistically significant.

#### 4.5.3 Domain of outcomes

The bias related to outcomes in the included studies was generally low. The definition of outcome variables consistently utilized guidelines or classification methods recognized by journals, and the intervals between the measurement of predictive factors and outcome indicators were established based on clinical expertise.

#### 4.5.4 Domain of statistical analysis

The bias in the domain of statistical analysis was high across the 32 studies. Twenty studies (16, 17, 20, 21, 23–28, 30, 33, 37–39, 41, 43–46) were conducted in a single center or limited to one geographic area. Six studies (27, 29, 31, 32, 37, 38) did not explicitly mention how missing data were addressed. Fifteen studies (19–21, 23, 24, 29–31, 35, 37, 39–42, 47) used random splitting methods for internal or external model validation, and 12 studies (16, 17, 19, 21–23, 25, 26, 38, 41, 42, 46) had insufficient sample sizes for model development and validation.

#### 4.5.5 Applicability assessment

The applicability assessment of the 32 included studies covered three aspects: study participants, predictive factors, and outcomes. All of the models included demonstrated good applicability across these three aspects, resulting in a generally high applicability assessment. The bias and applicability assessments included in the model are shown in **Table 3**.

**Table 3:** Assessment of bias and applicability in the included model.

Included literature	Bias assessment					Applicability Assessment			
	Study subjects	Predictive factor	Results	Statistical analysis	Overall bias risk	Study subjects	Predictive factor	Results	Overall applicability assessment
Nan	②	②	②	①	①	④	④	④	④
Wang et al.	②	②	②	①	①	④	④	④	④
Yang et al.	①	②	②	①	①	④	④	④	④
Hou et al.	①	②	②	①	①	④	④	④	④
Lu et al.	①	②	②	①	①	④	④	④	④
Zhang et al.	①	②	②	①	①	④	④	④	④
Gao	①	②	②	①	①	④	④	④	④
Zhang et al.	①	②	②	①	①	④	④	④	④
Cao et al.	①	②	②	①	①	④	④	④	④
Han et al.	①	②	②	①	①	④	④	④	④
Wu et al.	①	②	②	①	①	④	④	④	④
Miao et al.	①	②	②	①	①	④	④	④	④
Liao et al.	①	②	②	①	①	④	④	④	④
Hu et al.	②	②	②	①	①	④	④	④	④
Jiang et al.	①	②	②	①	①	④	④	④	④
Wang et al.	①	②	②	①	①	④	④	④	④
Dong et al.	①	②	②	①	①	④	④	④	④
Xi et al.	①	②	②	①	①	④	④	④	④
Xu et al.	①	②	②	①	①	④	④	④	④
Yang et al.	②	②	②	①	①	④	④	④	④
Dong et al.	①	②	②	①	①	④	④	④	④
Sun et al.	①	②	②	①	①	④	④	④	④
Wu et al.	②	②	②	①	①	④	④	④	④
Sun et al.	①	②	②	①	①	④	④	④	④
Zhou et al.	①	②	②	①	①	④	④	④	④
Gao et al.	①	②	②	①	①	④	④	④	④
Zhang et al.	①	②	②	①	①	④	④	④	④
Hui et al.	①	②	②	①	①	④	④	④	④
Gao et al.	①	②	②	①	①	④	④	④	④
Mu et al.	①	②	②	①	①	④	④	④	④
Liu et al.	①	②	②	①	①	④	④	④	④
Hui et al.	①	②	②	①	①	④	④	④	④

① = high risk of bias; ② = low risk of bias; ③ = high applicability risk; ④ = low applicability risk.

#### 4.5.6 Meta-analysis results

**Figure 4:**
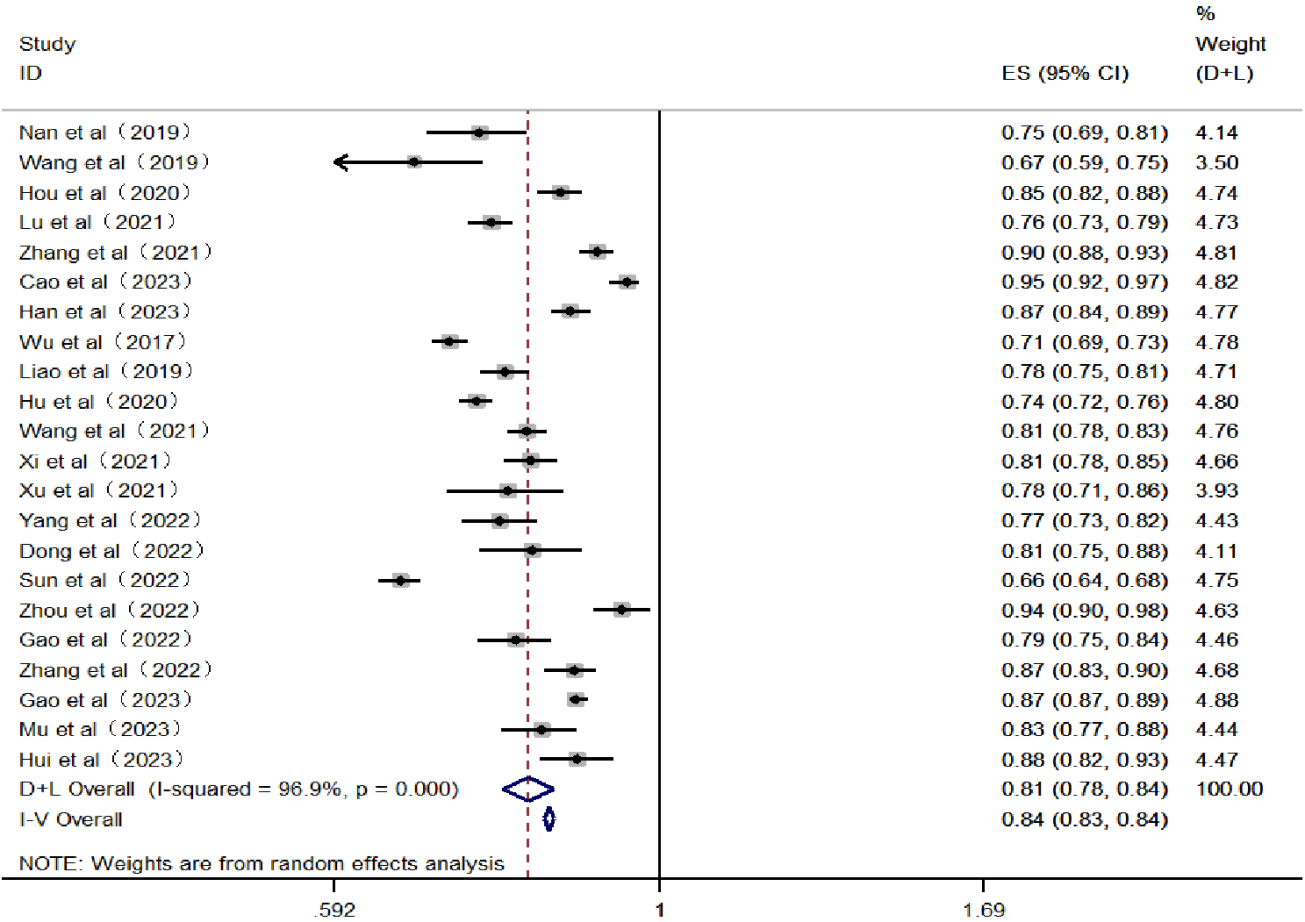
Forest plot of the area under the receiver operating characteristic curve for the risk prediction model.

The heterogeneity test results showed an *I* ^2^ = 96.9%, with *P* < 0.05, indicating significant heterogeneity among the included studies. Therefore, a random-effects model was utilized for the meta-analysis. After screening, it was found that 10 papers did not report AUC or 95% confidence intervals. Thus, the AUC of the remaining 22 papers was integrated into the meta-analysis. The results showed that the combined AUC was 0.810 (0.780–0.840), indicating good predictive performance. The specific forest plot is shown in **Figure 4**.

**Figure 5:**
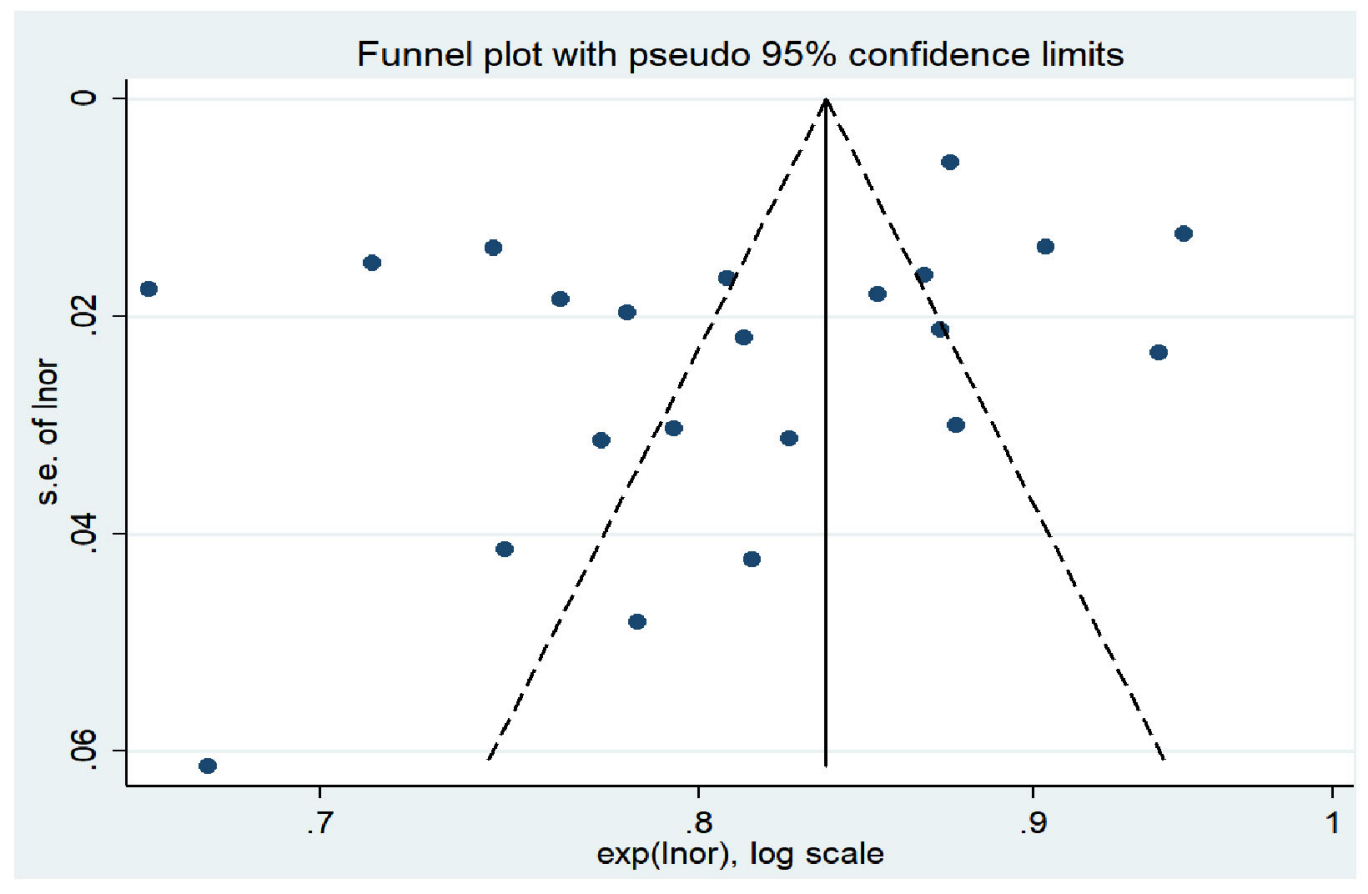
Funnel plot examination.

The results of Egger’s test showed a *t-*value of −1.74 and a *P*-value of 0.096 (*P* > 0.05), indicating that there was no significant publication bias in the study. In addition, the horizontal axis of the funnel plot represents the effect size of the study, while the vertical axis represents the sample size of the study (15). Studies with larger sample sizes are often located at the top of the funnel plot, near the mean effect size, while studies with smaller sample sizes are distributed at the bottom and sides of the funnel plot. Overall, the funnel plot presents a symmetrical funnel shape, with the points evenly distributed around the true value of the effect size, indicating a high level of consistency in the study results. This further suggests that there is no significant publication bias in this research. The funnel plot is shown in **Figure 5**.

## 5 Discussion

### 5.1 Hypertension, urine protein, creatinine, duration of diabetes, age, and fasting blood glucose are common predictive factors

This study systematically reviews existing risk prediction models for diabetic nephropathy in patients with type 2 diabetes in China. The aims are to evaluate the overall performance of these models and provide additional information to help select specific screening tools for prediabetic conditions. Most models include similar predictors, but there are differences in the weights assigned to each component. The study found that common predictors include hypertension, urine protein, creatinine, duration of diabetes, age, and fasting blood glucose. These factors are low cost and easily accessible.

Sixteen studies (20, 23, 24, 27, 31–34, 36–38, 40, 42, 44, 45, 47) consider hypertension a predictive factor for the risk of diabetic nephropathy in patients with type 2 diabetes. Sustained high blood pressure increases the burden on the kidneys. Compression of small arteries leads to arteriosclerosis of the renal arteries, thickening of the vascular wall, narrowing of the lumen, and increased glomerular filtration pressure and filtration rate, further causing increased albumin filtration. Renal lesions can also exacerbate hypertension, creating a vicious cycle, making hypertension a significant factor in the development of diabetic nephropathy (48). Advance et al. (49) found that lowering blood pressure can reduce vascular complications of diabetes by 10%. Therefore, for patients with diabetic nephropathy and hypertension, strict blood pressure control is crucial for protecting renal function in diabetic nephropathy patients. Optimizing blood pressure control can reduce the incidence of end-stage renal disease and adverse cardiovascular events. Therefore, patients should monitor their blood pressure under a doctor’s guidance as well as regularly review kidney function, urinary microalbumin, and other relevant indicators. If necessary, they should undergo individualized antihypertensive treatment under a doctor’s guidance. Dietary measures should focus on a low-salt diet and eating smaller, more frequent meals.

Thirteen studies (16, 18, 21, 24–26, 28, 30, 31, 33, 37, 39, 41) consider urine protein a predictive factor for the risk of diabetic nephropathy in patients with type 2 diabetes. When urine protein is positive, it indicates that protein is being lost through the urine, suggesting a disruption in the glomerular barrier function (50). Therefore, urine protein can provide valuable information for the diagnosis of diabetic nephropathy. Morphological studies by Said et al. (51) have shown that moderate increases in proteinuria are associated with glomerular and interstitial tubular damage in some diabetic patients. Therefore, patients should adhere to a low-salt diet, with a daily salt intake less than 6 g, avoiding salty pickles and preserved products and aiming for a bland diet. Moreover, moderate protein intake is a core and critical issue in the dietary management of diabetic nephropathy. Excessive protein intake can increase the glomerular filtration rate, promote thickening of the glomerular basement membrane, and increase kidney damage. Therefore, individuals with diabetic nephropathy should avoid high-protein diets and appropriately increase the proportion of high-quality protein in their diet.

Thirteen studies (16, 23, 24, 26, 28, 30–32, 34, 37, 40, 41, 46) have identified creatinine as a predictive factor for the risk of diabetic nephropathy in patients with type 2 diabetes. Creatinine, which is primarily filtered freely by the glomeruli, serves as a marker of kidney function impairment (26). When renal excretion is compromised, urates accumulate in the kidneys, leading to the buildup of creatinine and subsequent kidney damage (52). Early increases in creatinine levels are reversible; during this period, it is crucial to control blood sugar and reduce damaging factors. Additionally, medication, including traditional Chinese medicine or Western drugs, can help the body excrete excess metabolic products. In the case of treatment during the uremic phase, blood dialysis, peritoneal dialysis, and kidney transplantation are required.

Ten studies (17, 19, 22–25, 27, 44, 45, 47) have identified the duration of diabetes as a predictive factor for the risk of diabetic nephropathy in patients with type 2 diabetes. The duration of diabetes is a major factor in the occurrence of microvascular complications. As the duration of the disease increases, long-term dysregulation of glucose and lipid metabolism can cause irreversible damage to the kidneys. This leads to a continuous increase in urinary microalbumin, resulting in a gradual loss of glomerular function and the occurrence of end-stage renal events (33). Morton et al. (53) indicated that an extended duration of diabetes increases the risk of end-stage renal disease. For T2DM patients with a long history of the disease, it is important to take early measures such as lowering blood pressure, reducing lipid levels, adopting a low-salt diet, and improving kidney function to reduce the risk of developing diabetic nephropathy.

Twelve studies (17–19, 22, 28, 30–32, 36, 40, 45, 47) have identified age as a predictive factor for the risk of developing diabetic nephropathy in patients with type 2 diabetes. With increasing age, the reserve function of the kidneys gradually diminishes. After the age of 40, the glomerular filtration rate decreases by 1% with each additional year of age, meaning that between the ages of 50 and 90, the glomerular filtration rate can decrease by approximately 50%. Qiao et al. (54) found that the average age of the diabetic nephropathy patient group was 57.03 ± 12.425 years, which is approximately 6 years older than the average age of the non-diabetic nephropathy group. Therefore, for diabetic patients, greater attention should be paid to changes in kidney function as they age, including regular health check-ups. Additionally, a balanced diet, regular exercise, and stress relief are important. Many elderly individuals have chronic diseases requiring medication, but many drugs are nephrotoxic, so medication should be taken under the guidance of a professional physician.

Eight studies (21, 23, 32, 34, 35, 38, 42, 47) have identified fasting blood glucose as a predictive factor for the risk of developing diabetic nephropathy in patients with type 2 diabetes. Fasting blood glucose is the most fundamental marker of the progression of type 2 diabetes (34). Persistent hyperglycemia can induce mesangial expansion, leading to thickening of the basement membrane and hardening of the glomerular capillary walls, ultimately resulting in proteinuria and kidney damage. Furthermore, sustained high blood sugar levels cause the glomeruli to be in a state of high perfusion and filtration, leading to arteriosclerosis and changes in vascular permeability. This eventually results in progressive vascular proliferation, hyalinization, and intravascular thrombosis due to the extensive deposition of plasma proteins on the capillary walls (55). Lou et al. (56) found that for every 1 mmol/L increase in fasting blood glucose, the risk of proteinuria increases by 1.15 times. Therefore, medical professionals should make recommendations on the frequency of checks based on the patient’s blood sugar levels to ensure they are maintained within the target range. Dietarily, adherence to a low-sugar, high-fiber diet is advised, including consuming more fruits, non-starchy vegetables, whole grains, and legumes. Intake of saturated fats, processed meats, sweets, and sodium should be limited. Moreover, patients should follow their doctor’s advice on choosing the appropriate oral hypoglycemic drugs or insulin injections.

### 5.2 The model demonstrates good predictive power but still exhibits some bias

Generally, the performance metrics included in the models primarily report the sensitivity indicator AUC. Typically, the larger the AUC value, the better the model’s predictive ability. Among the 32 studies included in this research, 18 articles (16–18, 20, 21, 24, 26–30, 32, 35, 38, 42, 43, 45, 47) reported an AUC greater than 0.8. Furthermore, the results of the meta-analysis show a combined AUC value of 0.810 (0.780–0.840), indicating that the overall predictive performance of the existing risk prediction models for diabetic nephropathy in Chinese patients with type 2 diabetes is relatively good. Discrimination, calibration, and the clinical utility of the model are important parameters for evaluating predictive models. This study includes information on model calibration results and more from 22 articles (16–18, 21, 23, 24, 27, 28, 31, 33, 34, 36–42, 44–47). Moreover, only seven articles (21, 33, 36, 37, 39, 41, 44) conducted external validations, with the remaining articles lacking such verification; thus, the generalizability of these findings requires further validation. Conducting external studies on risk prediction models is crucial as it assesses the model’s generalizability and robustness across different populations and settings, thereby enhancing the model’s credibility and practical value. Additionally, external validation can reveal the model’s performance on new datasets, helping to identify potential overfitting and bias issues, thus promoting further optimization and improvement of the model (57). In terms of modeling methods, 21 articles (17–19, 21–25, 27, 30, 33, 34, 36–39, 42, 44–47) utilized logistic regression models, 6 articles (16, 20, 26, 29, 40, 41) used machine learning models, and 7 articles (23, 24, 28, 33, 34, 42, 45) employed LASSO regression models. Among these, 4 articles (31, 32, 35, 43) compared traditional models with machine learning models and found that the predictive performance and application effects of machine learning models were superior to those of traditional models. This indicates that prediction models based on machine learning have a promising future and high application value. Future researchers could develop risk prediction models based on machine learning within an interpretability framework to further improve the sensitivity, accuracy, and credibility of the models.

All 32 predictive models included in the analysis were assessed as having a high risk of bias, which limits their clinical applicability. The bias mainly stems from the type of study subjects; 27 studies (16–18, 21, 23–33, 35–47) were retrospective and deemed to have a high risk of bias. In terms of statistical analysis, 20 studies (16, 17, 20, 21, 23–28, 30, 33, 37–39, 41, 43–46) were conducted as single-center studies or only within the same region, 5 studies (27, 29, 31, 32, 37, 38) did not clearly mention how missing data were handled, and 15 studies (19–21, 23, 24, 29–31, 35, 37, 39–42, 47) used random split validation methods for model internal or external validation. Moreover, 12 studies (16, 17, 19, 21–23, 25, 26, 38, 41, 42, 46) had insufficient sample sizes for model development and validation. For future risk prediction model development and validation studies, it is recommended that researchers refer to the PROBAST standards to reduce bias in the research process and data analysis (58). Overall, although the models demonstrate moderate to good performance, the risk of bias remains high. Future improvements are needed in the type of studies conducted, the time interval between predictor assessment and outcome determination, the number of events, handling of missing values, and methods for selecting predictors.

In prediction model research, there is a direct relationship between the overall sample size and the number of participants with specific outcomes. In model development studies, sample size is often determined by the events per variable (EPV). An EPV of at least 10 to 20 is widely accepted as a standard to minimize overfitting (59). However, this study found issues with small sample sizes in the models. Therefore, it is necessary to use larger sample sizes for future model development and validation. A larger sample size yields more precise results, with smaller standard errors and narrower confidence intervals. It also helps ensure the model’s generalizability, meaning it can accurately predict outcomes in new datasets. Using a sufficiently large sample size allows researchers to capture more variability and potential confounders, leading to more accurate estimates of predictor effects. Additionally, an adequate sample size enhances the model’s stability and reliability. Thus, future research should strive to ensure sufficiently large sample sizes to support model development and validation (60). Furthermore, this study found that some models were developed using single-center studies, which may limit the models’ broad applicability. Future researchers should prioritize multicenter studies. Multicenter studies, especially those conducted across multiple regions, increase sample diversity and representativeness, reduce biases inherent in single-center studies, and improve the generalizability and extrapolability of the findings. They allow researchers to validate the interventions’ effectiveness across different cultural, environmental, and medical settings, promote interdisciplinary and international collaboration, and provide more comprehensive data for policy-making and public health practices. Despite challenges in coordination, data management, and cultural differences, multicenter studies are widely adopted for providing richer and more reliable scientific evidence.

A good prediction model should include not only the actual influencing factors but also statistical indicators that reflect the model’s effectiveness. However, these statistical indicators’ prediction and calibration estimates can vary significantly among different disease populations. When applying a prediction model to different populations, recalibration may be necessary. This means that when using the prediction model on new and diverse populations, we need to adjust the model to ensure its accuracy and reliability. This may involve reassessing the weights of risk factors in the model, updating statistical indicators, and performing necessary calibration or validation steps. Only then can we ensure that the prediction model can accurately predict the risk of diabetic nephropathy in other type 2 diabetes populations.

Due to the lack of established expert consensus or guidelines, there is no standardized method for the systematic evaluation of risk prediction models. The methods used in this study to summarize the reliability and clinical applicability of the included models are primarily based on existing research (61). Systematic evaluation of models is still in the exploratory stage, indicating the need for more prediction model studies. Although some models have undergone external validation, there is high heterogeneity when studying different regions or populations (62). Therefore, caution must be exercised, and thorough evaluation and adjustment must be conducted when applying risk prediction models to clinical settings or populations different from the original model (63). We recommend future studies conduct external validation of models in different centers and countries to ensure their generalizability. Additionally, we suggest adjusting these models based on local environments and study populations to achieve better predictive performance. Further research is needed to determine effective strategies for integrating models into clinical practice and to establish a unified method for systematically evaluating prediction models.

### 5.3 Implications for future research

In recent years, risk prediction models have become a hotspot in clinical research. Early screening and identification of high-risk populations help healthcare professionals develop targeted preventive measures, improve the utilization of medical resources, and enhance patient outcomes. This study’s methodological and quality evaluation of risk prediction models found that statistical analysis is a major factor contributing to the overall risk of bias in research. Future researchers could follow the PROBAST tool to standardize the reporting of study items. In terms of model-building methods, employing machine learning algorithms to establish disease risk prediction models has become a research focus in the fields of computing and medicine. Models built with machine learning algorithms outperform those created with logistic regression methods in predictive performance (64). Machine learning offers new possibilities for complex medical predictions, especially when dealing with multidimensional, non-linear, and interrelated data. Therefore, applying machine learning algorithms to improve model performance is an important direction for future research. Further exploration and application of machine learning models may enhance prediction accuracy and provide patients with more personalized risk assessment and management strategies (65). Regarding the presentation of models, converting models into probability calculation equations, simplified scoring system tables, line charts, or online calculators makes them more clinically applicable. Integrating these with electronic medical record systems meets healthcare professionals’ needs for model use. This approach supports the use of complex algorithms while reducing the workload of manual calculations, facilitating practical clinical operations. In terms of study design, risk prediction models should be constructed using prospective studies. Prospective studies can collect data in real time, reduce recall bias, and ensure a clear temporal sequence of data, thereby improving the accuracy and reliability of predictions. Such study designs can better control confounding factors and provide higher-quality evidence, helping to establish more precise causal relationships during model development. This ultimately enhances the model’s effectiveness and generalizability in practical applications (66). Since the models included in the literature have mostly not been validated with large samples externally, their extrapolation ability is somewhat hindered. Therefore, future studies could employ large-scale, multicenter research in China to externally validate models and explore the construction of predictive models best suited to the Chinese population. Moreover, healthcare professionals could leverage data mining technology and artificial intelligence to establish a risk prediction information platform, improving the accuracy of model predictions to enhance clinical work efficiency.

### 5.4 Limitations

One of the strengths of this study is that it systematically retrieved, screened, and extracted a wide range of data, including general characteristics, predictors, and study outcomes, to compare and summarize the performance of the models. However, in conducting the literature search and selection for this study, due to language restrictions, we only searched for literature in Chinese and English. While evaluating and analyzing the study results, we considered all available literature but did not account for regional biases, which may lead to potential publication bias. Also, considering the differences caused by genetics among different ethnic groups, this study only summarized existing research in China and provided information on predictive models. This may limit the generalizability of the study results to Western populations, and adjustments may be necessary when applying these models to different regions. Moreover, our focus was on the development, validation, and methodological description of the models, without evaluating their actual application effects. This is because application effects are influenced by multiple factors, such as data quality and the actual environment. To ensure the objectivity and reliability of our evaluation, the research focus was placed on the characteristics and performance of the models themselves. Future research could further assess the effectiveness and accuracy of these models in practical applications.

## 6 Conclusion

Through systematic search and selection, this study included 32 existing risk prediction models of diabetic nephropathy in patients with type 2 diabetes in China and systematically summarized the characteristics of these models. The results show that the overall efficacy of the models is good, but there is still a high risk of bias, and most lack external validation, so the clinical application effects of the models need further verification. In future research, scholars can rely on basic medical infrastructure and use big data to construct comprehensive, stable, and well-performing risk prediction models, providing further evidence-based data for the prevention of diabetic nephropathy in patients with type 2 diabetes.

## Data availability statement

The raw data supporting the conclusions of this article will be made available by the authors, without undue reservation.

## Author contributions

WX: Conceptualization, Formal analysis, Methodology, Project administration, Writing – original draft. QY: Funding acquisition, Project administration, Writing – review & editing. YZ: Investigation, Writing – original draft. QJ: Writing – original draft. YF: Writing – original draft.
